# HMGB1 and Extracellular Histones Significantly Contribute to Systemic Inflammation and Multiple Organ Failure in Acute Liver Failure

**DOI:** 10.1155/2017/5928078

**Published:** 2017-06-13

**Authors:** Runkuan Yang, Xiaoping Zou, Jyrki Tenhunen, Tor Inge Tønnessen

**Affiliations:** ^1^Department of Intensive Care Medicine, Tampere University Hospital, University of Tampere, 10 Bio Katu, 33014 Tampere, Finland; ^2^Department of Critical Care Medicine, University of Pittsburgh Medical School, 3550 Terrace Street, Pittsburgh, PA 15261, USA; ^3^Department of Emergencies and Critical Care, Oslo University Hospital, P.O. Box 4950 Nydalen, 0424 Oslo, Norway; ^4^Department of Gastroenterology, Drum Tower Hospital, Nanjing University Medical School, 321 Zhongshan Street, Nanjing 210008, China; ^5^Department of Surgical Science, Anesthesiology and Intensive Care Medicine, Uppsala University, 751 85 Uppsala, Sweden; ^6^Institute of Clinical Medicine, University of Oslo, Blindern, 0316 Oslo, Norway

## Abstract

Acute liver failure (ALF) is the culmination of severe liver cell injury from a variety of causes. ALF occurs when the extent of hepatocyte death exceeds the hepatic regenerative capacity. ALF has a high mortality that is associated with multiple organ failure (MOF) and sepsis; however, the underlying mechanisms are still not clear. Emerging evidence shows that ALF patients/animals have high concentrations of circulating HMGB1, which can contribute to multiple organ injuries and mediate gut bacterial translocation (BT). BT triggers/induces systemic inflammatory responses syndrome (SIRS), which can lead to MOF in ALF. Blockade of HMGB1 significantly decreases BT and improves hepatocyte regeneration in experimental acute fatal liver injury. Therefore, HMGB1 seems to be an important factor that links BT and systemic inflammation in ALF. ALF patients/animals also have high levels of circulating histones, which might be the major mediators of systemic inflammation in patients with ALF. Extracellular histones kill endothelial cells and elicit immunostimulatory effect to induce multiple organ injuries. Neutralization of histones can attenuate acute liver, lung, and brain injuries. In conclusion, HMGB1 and histones play a significant role in inducing systemic inflammation and MOF in ALF.

## 1. Background

Acute liver failure is defined as a clinical syndrome characterized by liver injury with evidence of coagulopathy and any degree of altered mental status in a patient without preexisting liver disease and duration of illness less than 26 weeks [1–3]. The etiology varies with geography. Hepatotrophic viruses are the most common cause of ALF in developing countries [4, 5]; drugs are the most common cause of ALF in the industrialized nations [4, 6]. The mortality of ALF is as high as 40–50%, and the cause of death in ALF includes brain herniation due to raised intracranial pressure (35%) and sepsis with multiple organ failure [3]. Liver transplantation is the only therapeutic intervention with proven survival benefit in patients with irreversible ALF [2]. ALF patients are prone to infection due to the immunologic defect and the high-dependency care they require [7]. Between 39% and 57% of ALF patients experience bacterial infection [8]. ALF has a high rate of infection with gram-negative enteric bacteria in animal model [9]. Gram-negative and gram-positive bacteria can elicit sepsis [7]. Infections and/or the resulting SIRS are important contributing factors that worsen hepatic encephalopathy (HE) [8]. ALF is associated with MOF and a high incidence of sepsis (35.7%), which contributes to 23.1% of the mortality [7]; however, the underlying mechanism is still not clear. Early inflammatory mediators (such as TNF-*α*, IL-6, and IL-1*β*) are certainly involved in the pathogenesis of ALF [10]; however, these early cytokines have limited clinical significance due to the narrow therapeutic window. In contrast, HMGB1 is a late mediator of lethal systemic inflammation [11], and HMGB1 has a prolonged therapeutic window as compared to those early inflammatory cytokines; circulating TNF-*α* and IL-6 are elevated for 5 days after the onset of sepsis, and serum HMGB1 levels are increased from day 7 until at least day 28 [12]. Emerging evidences indicate that HMGB1 is an important factor that links gut BT and sepsis, and extracellular histones are also important factors that significantly contribute to MOF in ALF. In this manuscript, we review the current understanding of HMGB1 and histones in ALF.

## 2. The Role of HMGB1 in ALF

### 2.1. HMGB1 is a Typical Alarmin

Endogenous danger-associated molecular patterns (DAMPs) are also known as alarmins, which signal cellular damage and activate the innate immune system [13]. Alarmins share the following features: (a) rapid release from cells in response to infection or tissue damage, (b) chemoattraction and activation of antigen-presenting cells, and (c) activation of innate and adaptive immunity [14]. HMGB1 is a typical alarmin [14].

### 2.2. Passive Release

HMGB1 can be passively released by necrotic/damaged cells or actively secreted by immunocompetent cells [15]. Under necrotic condition, contents of the cytosol are dispersed into the extracellular space due to cellular distress or damage [16]. In necrotic cells, HMGB1 dissociates from chromatin and is released from cells to trigger inflammation. However, HMGB1 remains bound to chromatin and fails to promote inflammation in cells undergoing apoptotic or programmed cell death, in which cytosolic contents are sequestered and not “seen” by innate immune cells [16].

### 2.3. Active Secretion

In response to proinflammatory stimuli such as LPS, HMGB1 can be actively secreted by immunocompetent cells. This active secretion occurs through a two-step process. First, HMGB1 is translocated out of the nucleus to the cytoplasm after JAK/STAT1-regulated hyper-acetylation of lysine residues located in the A and B box domains [17]. Once in the cytoplasm, HMGB1 is actively secreted [17]. The active secretion of HMGB1 follows a nonclassical, vesicle-mediated pathway [18].

### 2.4. Redox State and Extracellular Functions

HMGB1 contains three conserved redox-sensitive cysteines (C23, C45, and C106), and posttranslational modification (oxidation) of these cysteines determines the bioactivity of extracellular HMGB1 [19]. The cytokine-stimulating activity of HMGB1 requires C23 and C45 to be in a disulfide linkage, while C106 remains in a reduced state. This disulfide HMGB1 can bind and signal via TLR4/MD-2 complex to induce cytokine release in macrophage [19]. Moreover, binding of HMGB1 to TLR4 depends on reduced Cys106 [20]. If all three cysteines are in reduced form, this all-thiol HMGB1 has chemotactic activity. This form is present under basal conditions. Under inflammatory conditions, this all-thiol HMGB1 is released and forms a heterocomplex with the chemokine CXCL12 to interact with the chemokine receptor CXCR4 to promote cell migration, but no inflammatory cytokine secretion. The third form of HMGB1 occurs under a state of complete oxidation wherein each cysteine is fully oxidized to a sulfonyl form and is not associated with any biological function [19]. Hyperacetylation of HMGB1 shifts its equilibrium from a predominant nuclear location toward a cytosolic and subsequent extracellular presence. Hence, posttranslational modifications of HMGB1 determine its role in inflammation and immunity [19].

### 2.5. ALF Releases HMGB1 as a Signal of Inflammation

ALF occurs when the extent of hepatocyte death exceeds the hepatic regenerative capacity [21], and the mode of the cell death typically follows one of the two patterns: necrosis or apoptosis [21]. In a clinical trial, HMGB1 represents the circulating indicator of necrosis during acetaminophen hepatotoxicity; full-length and caspase-cleaved keratin-18 are circulating markers of necrosis and apoptosis; hyper-acetylated HMGB1 is a serum indicator of pyroptosis and immune cell activation [22, 23]. Elevations in plasma HMGB1 and keratin-18 can serve as mechanistic biomarkers to provide early and sensitive detection of acetaminophen-induced acute liver injury at first presentation to a hospital [22]. Increased total and acetylated HMGB1 and full-length keratin-18 are associated with worse prognosis during clinical acetaminophen hepatotoxicity [23]. HMGB1 can be released readily from necrotic or damaged cells to serve as a signal for inflammation [16, 24]. HMGB1 plays an important role in modulating inflammatory cascade in activated macrophages: HMGB1 stimulates macrophages to release TNF-*α* and IL-6 [20, 25]. HMGB1 is a potent mediator of systemic inflammation in sepsis [26].

### 2.6. HMGB1 Contributes to Multiple Organ Injuries

HMGB1 contributes to liver injury in experimental ischemia-reperfusion [27]. Exogenous HMGB1 injection can induce evident liver injury in mice [28]. HMGB1 impairs hepatocyte regeneration and blockade of HMGB1 improves hepatocyte regeneration in mice challenged with acetaminophen overdose [29]. A partly humanized anti-HMGB1 monoclonal antibody attenuates acetaminophen hepatotoxicity and postinjury inflammation in mice [30]. HMGB1 is released into the serum at early stage of D-galactosamine/LPS-induced ALF, and HMGB1 acts synergistically with TNF-*α* to promote the inflammatory liver injury; this detrimental effect can be reversed by monoclonal antibodies against HMGB1 and TNF-*α* [31]. Inhibition of sphingosine kinase-1 ameliorates experimental ALF by reducing high-mobility group box 1 cytoplasmic translocation in liver cells [32]. HMGB1 contributes to renal ischemia-reperfusion injury [33], sepsis-induced kidney injury [34], and severe acute pancreatitis-related kidney injury [35]. HMGB1 also significantly contributes to hemorrhagic shock-related acute lung injury (ALI) [36], hyperoxia-induced ALI [37], and severe acute pancreatitis-related ALI [38].

### 2.7. HMGB1 Contributes to Gut Mucosal Injury and Mediates Gut BT in ALF

Gut mucosal injury and intestinal BT in ALF is particularly important because the intestine is the biggest reservoir of bacteria in the body and leakage of bacteria or microbial products, notably LPS, from the lumen of the gut into the systemic circulation, leads to initiation or amplification of a systemic inflammatory response syndrome and multiple organ dysfunction syndrome (MODS). Therefore, the leaky gut is thought to be the “motor” that drives the development of MODS [39].

The serum HMGB1 concentrations are significantly increased in mice challenged with acetaminophen overdose [29, 40]. ALF patients also have high concentrations of circulating HMGB1, and the circulating HMGB1 levels are associated with disease severity [41, 42]. The circulating HMGB1 contributes to gut mucosal hyperpermeability and induces evident BT in experimental hemorrhagic shock and reperfusion [43], and exogenous HMGB1 injection can induce gut hyperpermeability and BT in mice [28]. Except from the circulating HMGB1, bile HMGB1 might also significantly contribute to intestinal mucosa injury and induce evident BT in ALF, because hepatitis E virus-related ALF is associated with significantly increased circulating LPS [44], and LPS injection reduces 40% of bile flow in rats [45]; adequate bile is required to maintain gut epithelial tight junction and intestinal bacterial homeostasis [46]; decreased gut luminal bile volume not only impairs intestinal tight junction but also changes intestinal bacterial homeostasis to facilitate BT [46, 47]. In addition, LPS injection (to normal animals) markedly increases bile TNF-*α* and HMGB1 levels, which can induce gut mucosal hyperpermeability and evident BT in mice [45], and this detrimental effect can be reversed by neutralization of the bile HMGB1 [45].

Experimental hepatotoxicity is associated with high incidence of gut derived gram-negative bacterial infection [9, 40]. Acetaminophen overdose can induce evident gut BT and severe intestinal mucosal injury in mice [40], and gut bacteria can adhere to the injured mucosa, which is necessary but not sufficient to induce gut BT [40], because blockade of HMGB1 reduces 85% of gut BT, but it does not decrease gut mucosal permeability in experimental acetaminophen overdose [40]. This indicates that BT is mediated (at least partly) by HMGB1 and BT is likely an active “transcellular” procedure in which HMGB1 is also needed.

### 2.8. BT Induces/Triggers Systemic Inflammation, Which Leads to MOF in ALF

Gut BT (or gut-derived LPS) induces/triggers systemic inflammation in critical illness [48, 49]. SIRS can lead to MOF in ALF [7]. Sepsis is a typical example of SIRS triggered by infection [50]. Therefore, HMGB1 seems to be an important factor that links BT and SIRS in ALF.

## 3. The Role of Extracellular Histones in ALF

### 3.1. ALF Patients/Animals Have High Concentrations of Circulating Histones

ALF has a large number of hepatocyte death [21]. The necrotic tissue/the dying hepatocytes release HMGB1 and histones [50], which can contribute to the high concentrations of circulating histones in animals challenged with concanavalin A and acetaminophen overdose to induce two different acute fatal liver injury models [50]. Circulating histones are significantly increased in ALF patients and in patients with HBV-related acute-on-chronic liver failure, and the levels of circulating histones are correlated with disease severity and mortality [51, 52]. ALF patients also have elevated plasma histone-associated DNA levels [53]. DAMPs activate innate immune cells in the liver and the circulation, subsequently leading to tissue inflammation and SIRS [53, 54].

### 3.2. Extracellular Histones Contribute to Multiple Organ Injuries in ALF

Histones are important structural elements of nuclear chromatin and regulate gene transcription [54]; however, extracellular histones are cell toxic to host cells [50, 54] and elicit immunostimulatory effect that can induce multiple organ injuries [50, 53–55]. Circulating histones exacerbate inflammation in mice with ALF [56]. The sera (containing high levels of histones) from ALF patients can induce L02 cell (hepatocyte) death and stimulate U937 cells (monocytes) to release inflammatory cytokines; these detrimental effects can be abolished by non-anticoagulant heparin that can bind histones, suggesting that circulating histones might be the major mediators of systemic inflammation and cellular injury in patients with ALF [51]. The circulating angiopoietin-2 levels, a marker of tissue endothelial dysfunction and leakage, are markedly increased in ALF patients [53], and this might be due to the toxicity of extracellular histones in ALF patients. Extracellular histones contribute to acute fatal liver injury via TLR 2 and TLR4 receptors, and neutralization of histones can ameliorate fatal liver injury in mice [50]. The levels of circulating histones are significantly higher after liver ischemia/reperfusion, the endogenous histones function as alarmins in sterile inflammatory liver injury through toll-like receptor 9, and neutralization of histone significantly protects against injury [57]. Extracellular histones can induce microvascular endothelial injury, and the TLR2/4-mediated inflammation leads to acute tubular necrosis in experimental acute kidney injury [58, 59]. Extracellular histones can injure endothelial cells to cause microvascular thrombosis and hemorrhage in experimental acute lung injury [60]. Histones also contribute to experimental acute brain injury, and neutralization of histones can reduce the infarct size [61]. Histone H4 and increased circulating neutrophil extracellular traps (NETs) can activate platelets; this may cause microvascular thrombosis in sepsis [50, 55, 59, 62]. HMGB1 may induce the same biological response [63]. Extracellular histones kill endothelial cells and are one of the major mediators of death in sepsis [55].

## 4. Conclusions

HMGB1 and extracellular histones play a significant role in inducing systemic inflammation and MOF in ALF; neutralization of HMGB1 and histones may present a novel therapy to treat ALF.

A graphical abstract is provided as the Supplementary material available online at https://doi.org/10.1155/2017/5928078.

## Supplementary Material

In acute liver failure, the hepatocyte death releases HMGB1 and histones. HMGB1 contributes to multiple organ injury and mediates gut BT, BT triggers systemic inflammation that can lead to multiple organ injury. Histones contribute to multiple organ injuries by injuring endothelial cells and activating platelets to induce vascular thrombosis.

## Abbreviations

ALF: Acute liver failure
MOF: Multiple organ failure
SIRS: Systemic inflammatory response syndrome
BT: Bacterial translocation
HMGB1: High mobility group box 1
NETs: Neutrophil extracellular traps
DAMP: Damage-associated molecular pattern
TLR: Toll-like receptor
APAP: Acetaminophen
MODS: Multiple organ dysfunction syndrome
MD-2: Myeloid differentiation factor 2
LPS: Lipopolysaccharide
D-GalN: D-galactosamine
Sphk1: Sphingosine kinase 1.
