# Supplementary material for: HMGB1 and Extracellular Histones Significantly Contribute to Systemic Inflammation and Multiple Organ Failure in Acute Liver Failure
Source: Mediators Inflamm. 2017 Jun 13;2017:5928078. doi: 10.1155/2017/5928078 (PMC5485317; doi:10.1155/2017/5928078)
Supplement: Supplementary file 1 — In acute liver failure, the hepatocyte death releases HMGB1 and histones. HMGB1 contributes to multiple organ injury and mediates gut BT, BT triggers systemic inflammation that can lead to multiple organ injury. Histones contribute to multiple organ injuries by injuring endothelial cells and activating platelets to induce vascular thrombosis. [file 5928078.f1.pptx]

## Slide 1
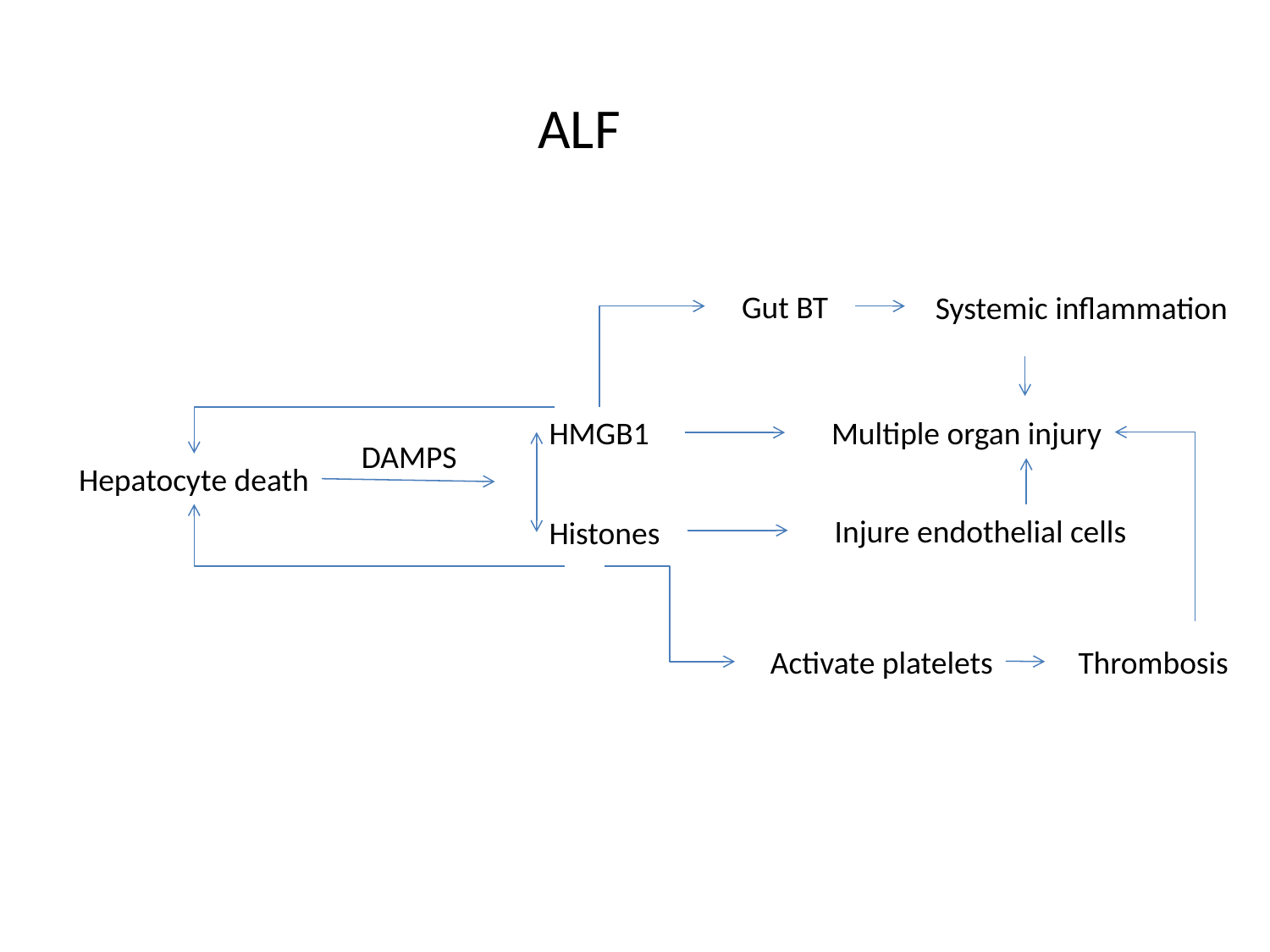

ALF
Gut BT
Systemic inflammation
HMGB1
Multiple organ injury
DAMPS
Hepatocyte death
Injure endothelial cells
Histones
Activate platelets
Thrombosis
